# Correction: Correction: Effectiveness and satisfaction of mindfulness-based cognitive therapy for children on anxiety, depression, and internet addiction in adolescents: Study protocol for a randomized control trial

**DOI:** 10.1371/journal.pone.0336877

**Published:** 2025-11-14

**Authors:** The PLOS One Staff

There is an error in the correction published on September 2, 2025. Masume Bakhtiari & Mojtaba Habibi Asgarabad contributed equally to the manuscript as the first co-authors.

The publisher apologizes for this error.
